# Emerging therapeutic approaches for peritoneal metastases from gastrointestinal cancers

**DOI:** 10.1016/j.omton.2024.200767

**Published:** 2024-01-29

**Authors:** Aleksandra Sikora, Kevin M. Sullivan, Sean Dineen, Mustafa Raoof, Aleksandra Karolak

**Affiliations:** 1 Department of Medicine, Medical University of Warsaw, 02-091 Warsaw, Poland; 2 Division of Surgical Oncology, Department of Surgery, City of Hope National Medical Center, Duarte, CA 91010, USA; 3 Department of Gastrointestinal Oncology, H. Lee Moffitt Cancer Center and Research Institute, Tampa, FL 33612, USA; 4 Department of Cancer Genetics and Epigenetics, City of Hope National Medical Center, Duarte, CA 91010, USA; 5 Department of Machine Learning, H. Lee Moffitt Cancer Center and Research Institute, Tampa, FL 33612, USA

**Keywords:** MT: Regular Issue, peritoneal metastases, metastatic gastrointestinal cancer, intraperitoneal therapy, drug delivery, emerging treatments

## Abstract

Peritoneal metastases from gastrointestinal malignancies present difficult management decisions, with options consisting primarily of systemic chemotherapy or major surgery with or without hyperthermic intraperitoneal chemotherapy. Current research is investigating expanding therapeutic modalities, and the aim of this review is to provide an overview of the existing and emerging therapies for the peritoneal metastases from gastrointestinal cancers, primarily through the recent literature (2015 and newer). These include the current data with systemic therapy and cytoreduction with hyperthermic intraperitoneal or pressurized intraperitoneal aerosol chemotherapy, as well as novel promising modalities under investigation, including dominating oncolytic viral therapy and adoptive cellular, biologic, and bacteria therapy, or nanotechnology. The novel diverse strategies, although preliminary and preclinical in murine models, individually and collectively contribute to the treatment of peritoneal metastases, offering hope for improved outcomes and quality of life. We foresee that these evolving treatment approaches will facilitate the transfer of knowledge and data among studies and advance discovery of new drugs and optimized treatments for patients with peritoneal metastases.

## Graphical abstract

**Figure:**
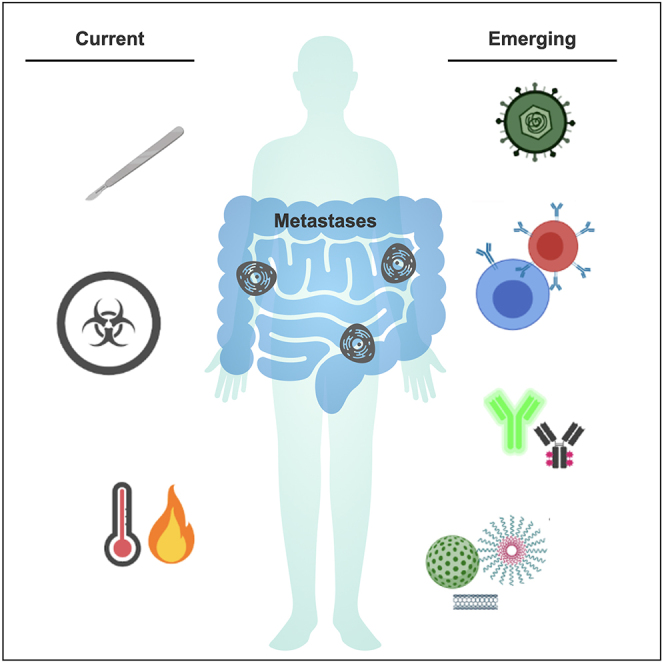

## Teaser

Sikora and colleagues discuss the existing and emerging therapies for peritoneal metastases (PM) through the recent literature (2015 and newer). They overview novel strategies, which individually and collectively contribute to the treatment of PM, offering hope for improved outcomes and quality of life.

## Introduction

The peritoneal cavity is a common metastatic site for gastrointestinal (GI) cancers. Because of the high propensity for early metastatic progression of the primary tumors, 1 out of 3 individuals with metastatic GI cancer develops peritoneal metastases (PM). The rates of PM occurrence also vary among primary tumor sites of GI cancers;^1^ gastric and colon cancers are the most common GI cancers to develop PM, because 14% of all gastric and 7% of all colon cancers will develop PM.^1^^,^^2^^,^^3^ Colorectal cancer (CRC) is the second most common cause of cancer-related deaths in the United States,^4^ and gastric cancer ranks as the fourth leading cause of cancer-related death worldwide.^5^ Over 20% of advanced (stage IV) CRC and over 60% of advanced (on average, 30% of all^3^) gastric cancers develop PM resistant to systemic chemotherapy.^6^ PM resulting from other primary malignancies, such as the pancreas, appendix, or small intestine, occur less frequently overall. In addition, approximately 5% of cases of PM are of unknown origin.^7^

**Figure 1:**
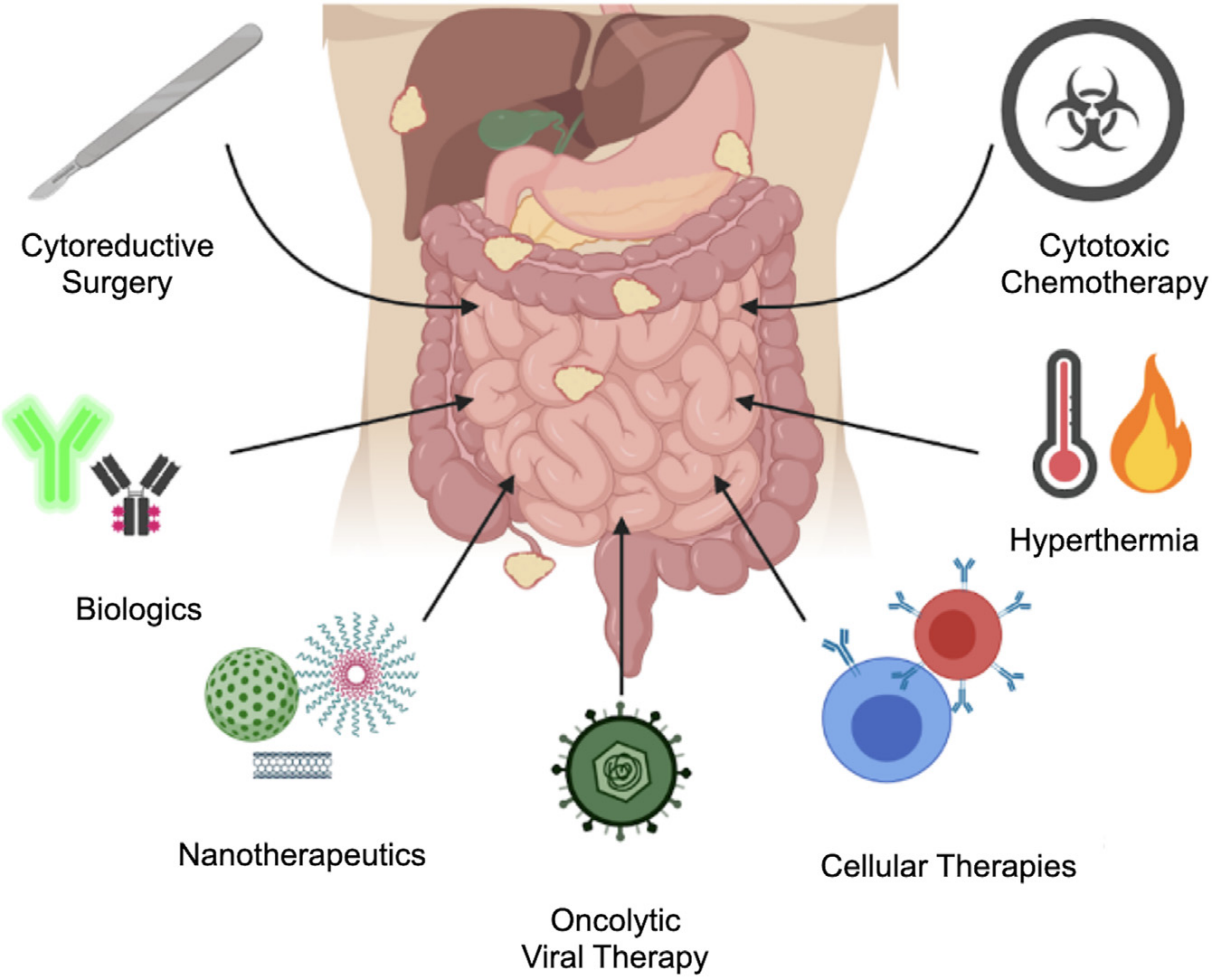
Current and emerging treatment approaches in peritoneal metastases

Compared to other sites of metastases, patients with carcinomatosis have worse survival,^6^ which varies among primary tumor sites. The mean survival of patients with CRC and PM was reported to be 6.9–8.5 months, pancreas cancer was 2.4–2.9 months, and gastric cancer was 2.2–6.5 months.^8^^,^^9^ Despite the increase in multimodal management of metastatic cancer, survival outcomes of PM patients remain dismal, likely because of several reasons, including the following: (1) PM remains undetected on radiographic imaging until symptomatic disease has developed, and patients are not candidates for surgery; (2) approved systemic therapies for metastatic GI cancers have limited nondurable efficacy for PM;^10^ and (3) the rapid decline in performance status precludes approved systemic therapy options. Compared to patients with other sites of metastases, patients with PM have a worse quality of life, which is caused by developing ascites, bowel obstructions, and malnutrition. Therefore, novel therapies that are tolerable and effective for symptomatic patients with PM are urgently needed to improve overall outcomes for this disease process (Figure 1).

### Current therapeutic approaches and challenges

The surface area of the peritoneum in its entirety is nearly 2 m^2^ in adults, similar in surface area to the skin. However, the blood supply to the peritoneum represents only ∼2% of cardiac output (Solass Pleura Peritoneum, 2016^11^). This has important challenges for the transport and delivery of therapeutic agents into this metastatic site. Naturally involved in the exchange of nutrients, the peritoneal cavity also provides an immune barrier that is able to respond to various pathogens through the transfer of immune cells. However, Li and Guo have noted that the role of peritoneal cells on tumor dissemination to peritoneum remains controversial, with evidence suggesting inhibiting or promoting effects.^12^ PM diagnosis and quantification is commonly assessed by cross-sectional imaging and laparoscopy to determine the extent of peritoneal involvement. This staging examination also allows for the selection of some patients for cytoreductive surgery (CRS), with or without the addition of intraperitoneal (IP) chemotherapy. For patients who are not candidates for CRS, systemic therapy or best supportive care is the typical choice.

#### Systemic therapies

Chemotherapy has improved survival for most individuals with metastatic GI cancers, yet its impact on the survival among patients with PM has remained significantly low. Current systemic therapies approved for metastatic GI cancers are less effective for PM, in part because of the hindered penetration of drugs into the peritoneum, owing to the plasma-peritoneal barrier, peritoneal symptoms limiting chemotherapy, disease symptomatic progression, and intolerance to the treatment plan. Nonetheless, systemic chemotherapy (or best supportive care) is the recommended guideline.

The comprehensive summary of all of the current chemotherapeutic approaches across GI cancers can be found under the National Comprehensive Cancer Network guidelines (https://www.nccn.org/guidelines/). For patients with advanced gastric cancer, the first-line therapy typically consists of fluoropyrimidines, such as 5-fluorouracil (5-FU) and platinum-based agents. For the resectable gastric cancers, neoadjuvant application of epirubicin, cisplatin, and 5-FU followed by surgery (this study was registered at Clinicaltrials.gov: NCT93793971) showed improved survival compared to postoperative adjuvant therapy.^6^ Other combinations, including 5-FU, leucovorin, oxaliplatin, and docetaxel, also improved the outcomes, although these therapies led to significant side effects.^13^ In the metastatic setting, the first-line systemic chemotherapy consists of fluoropyrimidine and oxaliplatin chemotherapy,^14^^,^^15^ which can be further supported by immunotherapy (nivolumab in the CheckMate 649 trial^16^). In the second-line setting for unresectable gastric cancer, ramucirumab and paclitaxel were superior to paclitaxel alone in the RAINBOW trial.^17^ The clinical trial NCT01041404, using trastuzumab and chemotherapy, reported improved outcomes and survival for human epidermal growth factor receptor 2-positive (HER2^+^) gastric cancer.^18^

Targeted therapies are also being explored—for example, therapy directed at claudin 18.2 (CLDN18.2), a gastric-specific form of the tight-junction protein CLDN18. Zolbetuximab is a monoclonal antibody that targets CLDN18.2 and was recently reported in Phase III clinical trials to show efficacy for patients with, for example, CLDN18.2^+^ HER2^−^ gastric cancer. The overall survival (OS) and progression-free survival was improved in combination with either FOLFOX^19^ or CAPOX^20^ compared to FOLFOX or CAPOX alone.

Systemic therapy is associated with a low survival rate also for patients with PM from CRC and appendiceal cancers; thus, combined therapies have drawn attention over the years.^21^ The current first-line chemotherapy is either FOLFOX^22^ or FOLFIRI.^23^ Patients with CRC-PM treated with modern chemotherapy from the CAIRO1 and CAIRO2 trials have a reduced median OS of 10.4–15.2 months compared to patients without PM who had a median OS of 17.3–20.7 months.^24^ Evidence for some efficacy of systemic chemotherapy in CRC metastatic to the peritoneum comes from the CAIRO6 trial,^25^ which demonstrated an objective radiologic response rate among 28% of the patients who underwent neoadjuvant chemotherapy followed by surgery and an objective major pathology response rate of 38%. However, regardless of chemotherapeutic regimen used (oxaliplatin vs. irinotecan), patients with PM have worse survival compared to patients with non-PM disease.^26^ The addition of bevacizumab, a vascular endothelial growth factor (VEGF) monoclonal antibody, to FOLFOX was shown to improve progression-free survival and OS^27^ among patients with metastatic CRC. For *KRAS* wild-type tumors or left-sided colon tumors, the epidermal growth factor receptor antagonist antibodies cetuximab^28^^,^^29^ or panitumumab^30^^,^^31^^,^^32^ can be added to first-line chemotherapy. In the second-line setting for patients with *BRAF* V600E– mutated tumors, encorafenib, binimetinib, and cetuximab were shown in the BEACON trial to be effective with respect to the response rate and OS.^33^ Data for the efficacy of chemotherapy in high-grade appendiceal carcinoma and small bowel cancer are, however, largely extrapolated from studies of CRC.

In hepatobiliary and pancreatic cancers that are unresectable or metastatic, systemic chemotherapy is the recommended treatment modality. For hepatocellular carcinoma, the first-line therapy consists of atezolizumab and bevacizumab^34^ or tremelimumab-actl and durvalumab,^35^ and the second-line therapy is usually regorafenib^36^ or cabozantinib.^37^ For metastatic biliary tract cancers, durvalumab, gemcitabine, and cisplatin^38^ are the first-line chemotherapy, and FOLFOX is the second line.^39^ FOLFIRINOX^40^ and gemcitabine plus albumin-bound paclitaxel^41^ are the mainstays of systemic chemotherapy for pancreatic cancer. However, for each of these therapies, the efficacy, specifically for peritoneal carcinomatosis, is either not studied or not reported and therefore unknown.

#### Multimodal therapies

Despite the development of improved systemic therapies, PM from GI cancers remains resistant because of the plasma-peritoneal barrier formed by the tissue that surrounds the peritoneum^42^^,^^43^ and tumor-intrinsic properties. The kinetics of drug absorption and distribution across the peritoneum vary across agents and patients. Consequently, a multimodal strategy that combines surgery with systemic or regional chemotherapy results in optimal outcomes.

The addition of heated intraperitoneal chemotherapy (HIPEC) to CRS appears to be beneficial in the appendiceal histology; the retrospective propensity score-matched study of almost 2,000 patients showed improved OS without increased morbidity in CRS/HIPEC compared to CRS alone.^44^ Perioperative systemic chemotherapy and CRS (removing all visible peritoneal disease) are becoming more established as standard treatments for patients with CRC or high-grade appendiceal cancer. Even though 80% of patients will relapse after multimodal treatment, the addition of surgery is thought to prolong life and preserve the quality of life. The addition of HIPEC at CRS in the setting of CRC was investigated in a randomized clinical trial (PRODIGE 7). This study failed to demonstrate the benefit of HIPEC (oxaliplatin for 30 min, 42°C at 400 mg/m^2^). However, the OS of all of the patients was higher than expected (41 months), suggesting that CRS is beneficial for survival. The value of HIPEC with other chemotherapeutics, such as mitomycin C, is unclear and remains to be investigated in future studies.

For appendiceal cancer, based on retrospective studies, HIPEC with mitomycin C is considered standard practice. A randomized trial is unlikely to be performed because of the rarity of appendiceal cancer. For patients with unresectable PM, new treatment delivery options, such as pressurized intraperitoneal aerosol chemotherapy (PIPAC), are undergoing clinical trials. Initial studies have established safety and early efficacy. However, large prospective and randomized trials are needed to establish PIPAC over systemic chemotherapy alone.

For gastric cancer, regional chemotherapy and CRS are not standard.^45^ Several ongoing clinical trials are testing the multimodal management of gastric cancer PM. A retrospective case-control study demonstrated that CRS with HIPEC was more favorable than CRS alone in PM from gastric cancer. This study reinforced data from other studies, indicating that patients with small-volume PM that can be completely resected are the most likely to benefit.^46^ However, this question needs to be addressed in future randomized trials. Another approach of iterative, minimally invasive laparoscopic HIPEC without CRS has been investigated. In this approach, laparoscopic HIPEC is repeated multiple times, along with systemic therapy. If the PM are completely treated (negative cytology with Peritoneal Cancer Index 0), then gastrectomy is performed. Repeated laparoscopic HIPEC for gastric cancer PM demonstrated the ability to downstage PM and undergo gastrectomy with median OS from diagnosis of metastatic disease of 24 months, but with a relatively high complication rate.^47^^,^^48^ The PHOENIX-GC trial^49^ was a randomized trial that evaluated the benefit of normothermic-intraperitoneal (IP) paclitaxel delivered via port along with systemic therapy over systemic therapy alone. Hydrophilic taxanes (examples include docetaxel or paclitaxel) show improved pharmacokinetic characteristics, gradual absorption, and extended retention in the peritoneal cavity, bringing advantages to repeated IP chemotherapies. Normothermic-IP chemotherapy continues to be explored with a different systemic therapy regimen (STOPGAP trial; this study was registered at Clinicaltrials.gov: NCT04762953). PIPAC is also being investigated for gastric cancer PM.^50^^,^^51^ Three regimens have been used in early-phase clinical trials: cisplatin with doxorubicin, oxaliplatin, and paclitaxel. The heterogeneity of the PIPAC studies with variable concurrent systemic treatments makes it challenging to draw conclusions regarding efficacy; thus, the randomized studies are needed to establish the role of PIPAC in gastric cancer.

To date, the benefit of regional therapy (HIPEC, normothermic-IP chemotherapy, or PIPAC) remains unproven. Although there is a pharmacokinetic advantage to delivering agents directly into the peritoneal cavity, the effectiveness of IP chemotherapy depends on the homogeneous delivery of a drug to the peritoneum and infiltration of peritoneal tumors. In addition to improved surgical or delivery systems, drug modifications and enhancement of drug penetration alongside improved representative models (organoids) and molecular imaging should be investigated to better manage the PM not only from CRCs.^52^ The underlying mutational landscapes, immune profile, and tumor microenvironment could further benefit individual patients and metastatic subtypes. Despite that molecular analyses of cancer have evolved in recent years, the genetic alterations and biomarkers in PM remain largely unknown, limiting treatment optimization, largely caused by high-heterogeneity GI cancers.^53^^,^^54^

### Emerging therapies

Considering the insufficient results of the current treatment of PM, new or improved treatments are needed.^55^^,^^56^ In this section, we report novel treatment strategies, including oncolytic viral approaches, , cellular therapies, nanotherapeutic agents, and biologics.

#### Oncolytic viral therapy

**Figure 2:**
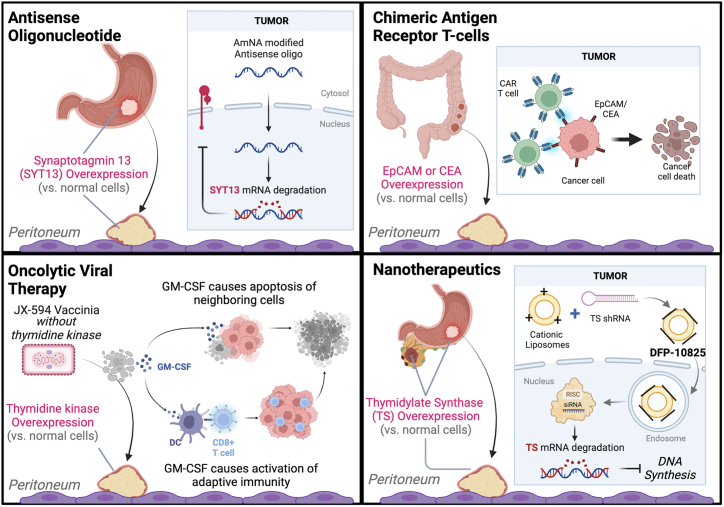
Mechanisms of the emerging treatment approaches in peritoneal metastases

Oncolytic viruses can be natural or modified viruses that selectively replicate and kill cancer cells without damaging normal tissues. Viral therapy can reduce tumor burden in several ways, including direct oncolysis of tumor cells and inhibition of angiogenesis mechanisms. Recently, oncolytic-virus therapy has become recognized as a promising targeted treatment for GI cancers because of its potential to synergize with chemotherapy and radiotherapy, as well as its potency to be used in the single-agent viral therapy.^57^ Several viruses have been investigated for the treatment of PM from GI cancers, and the leading families include *Herpesviridae*, *Adenoviridae*, *Rhabdoviridae*, and *Poxviridae*. Selection of these viruses has been driven by their ability to exploit and enhance the host’s antitumor immunity as well as their capacity to be genetically engineered to express tumor-destructive factors.^58^ Few have reached the clinical trial, with their efficacy still being evaluated on the preclinical level. Oncolytic viral therapy faces the main problem of neutralizing antibodies that circulate in the blood and reduce the efficacy of the oncolytic virus.^59^ In addition, tumor and stroma features can prevent the penetration of oncolytic viruses, causing resistance. Here, we discuss the most promising oncolytic viral approaches since 2015, with their major advantages and disadvantages summarized in Table 1 and example mechanisms illustrated in Figure 2.

**Table 1:** Advantages and disadvantages of the emerging therapies for peritoneal metastases and examples of mechanisms behind them

Therapy	Advantages	Disadvantages	Examples
**Virus therapy**			

Herpesvirus	Surface glycoproteins can be altered to target specific cellular receptors Replication can be controlled with herpesvirus-specific drugs Favorable cytopathic effect and replication potential in several human gastric cell lines	Risk of neuroinvasion Limited efficacy after systemic delivery	HSV-1, G47Δ^60^
Adenovirus	Genetic stability and low integration of DNA into host genomes Produced easily at high concentrations	Limited tumor targeting and efficacy due to high levels of neutralizing antibodies	OBP-301^61^ Combination of OBP-401 and paclitaxel^62^ OBP-702^63^ Ad/TRAIL-E1^64^ Ad-hARF^65^^,^^66^
Rhabdovirus	Ability to break local immunosuppression Lack of preexisting neutralizing antibodies in human populations Short replication cycle No cell-transformation nor immune-mediated pathogenesis in the host Higher selectivity for tumor cells	Ability to infect the CNS (cause neuropathology) High doses may cause a massive immune and inflammatory response that may be harmful or lethal	VSV^67^^,^^68^ M51R VSV^69^^,^^70^
Vaccinia virus	Direct cell lysis Interference with tumor angiogenesis and reduction of blood flow to tumor cells resulting in hypoxia	Limited efficacy after systemic delivery (neutralizing antibodies) Ability to replicate in a wide range of human cells and affect normal and untargeted cells	vvDD-IL15-Ra^71^^,^^72^ mJX-594 and anti-VEGFR2 or checkpoint inhibitors^73^

**Other emerging therapies**			

Bacteria	Low cost, immunostimulatory activity, biosafe strains available, low incidence of side effects, tendency to colonize the tumor microenvironment strictly Natural preference for tumor tissues	Potential toxicity; requires attenuation The host’s immune response can eliminate the introduced bacteria Compared to small-molecule drugs, live bacteria require complex production	Salmonella typhimurium^74^ *Salmonella*-IL-2^75^
Cellular therapy	CAR T cells show the greatest potential for improved survival Target specific surface antigens Break/remodel tumor microenvironment barrier and prevent immunosuppression Control cytolytic activity and temporary CAR expression, thus decreasing potential toxicity IL-2 enhance CAR T cell number and function, increase serum IFN-γ levels, and improve CEA responses IL-10 blockade increases CAR T migration, proliferation, and cytotoxicity	Risk of adverse events of cytokine release syndrome and on-target/off-tumor effects Potential to develop anti-CAR antibodies Challenges in developing and optimizing combination therapies Safety concerns Systemic IL-2 cause more severe but manageable adverse events IL-10 activates antigen-experienced T cells and induces immune-mediated tumor regression Risk of adverse events of cytokine release syndrome and on-target/off-tumor effects	Anti-EpCAM–CAR Ts^76^ CEA-CAR Ts^77^ CEA-CAR Ts combined with IL-2 or IL-10^78^^,^^79^ CEA-CAR Ts and MDSC and Treg antibodies^80^
Biologics and other	Support chemotherapy outcomes by improving drug delivery and inhibiting metastatic factors Reduction of microvessel density and VEGF levels No apparent side effects Improved drug penetration and delivery to tumors Disruption of the key stages of peritoneal metastatic progression (SYT13) Acceptable toxicity and minimal off-target effects (AmNA-modified anti-SYT13 ASOs) ASOs have extended half-life time and lipophilicity to confer resistance to peritoneal absorption Enhance efficacy of 5-FU Reduced tumor microvessel density and VEGF levels Reduced need for CRS with further optimization of administration schedules	Often lack the route and duration of administration In cases, poor delivery to solid cancer tissues Insufficient disease specificity Systemic toxicity Route and duration of administration of siSYT13 were not determined ASOs are vulnerable to endogenous nucleases, have poor delivery to solid cancer tissues, and have insufficient disease specificity Potential toxicity due to collagen breakdown in healthy tissues Insufficient data on the efficacy and toxicity of the iRGD system under conditions mimicking clinical protocols	siSYT13, AmNA- modified anti-SYT13 ASOs, SYT8-siRNA^81^^,^^82^^,^^83^ Adv-shPGK1^84^ ZnPPIX^85^ Collagenase^86^ iRGD^87^
Nanotherapeutics and hydrogels	A wide range of applications Small size allowing for enhanced drug delivery and penetration into peritoneal tumors Ability to modify surfaces for delivery and targeting Limited distribution to blood circulation prevents severe side effects Selective accumulation in disseminated tumors High biocompatibility and chemical stability No biochemical and histological abnormalities or structural changes Induce of long-term immune memory Adjuvant nano-formulations activate dendritic cells	Larger formulations (up to 1,000 nm) limit efficacy Optimizing drug-release kinetics and dosage can be challenging Unknown mechanism for selective accumulation in peritoneally disseminated tumors Incomplete eradication of metastases, development of malignant ascites Strict concentration of magnetic nanoparticles required for generating sufficient heat for effective ablation Insufficient for inhibition of established distant tumors	DFP-10825^88^ Carboxydextran-coated SPIONs in combination with an alternating magnetic field^89^ Ablation combined with PLGA-R837, PLGA-MPLA, or anti-CTLA-4^90^

Herpes simplex virus (HSV) belongs to the *Herpesviridae* family and has a large double-stranded DNA (152 kb), ∼30 kb of which encodes genes that are not essential for viral infection.^91^ Therefore, the genome of HSV is susceptible to modification by genetic recombination, which makes it an attractive agent for oncolytic viral therapy. Given that *in vitro* administration of the triple-mutated, third-generation oncolytic HSV-1 G47Δ has shown both a favorable cytopathic effect and replication potential in several human gastric cell lines, the examination of its therapeutic potential in clinic seems an attractive opportunity^60^ (by modifying immunosuppressive cells [regulatory T cells and tumor-associated macrophages], G47Δ facilitated the transport of effector immune cells into the tumor microenvironment in glioblastoma^92^). Mouse models with subcutaneous tumors treated intratumorally with G47Δ showed significantly inhibited growth caused by increasing expression of immunostimulatory molecules and enhancing M1-macrophage polarization and infiltration. More important, a low dose of G47Δ inhibited tumor growth as effectively as a high dose, reflecting good replication capability demonstrated by *in vitro* studies.^60^ In addition, G47Δ markedly decreased M2 macrophages in subcutaneous tumors while increasing M1 macrophages and natural killer cells, which can be necessary for the efficacy of oncolytic viral therapy.^92^^,^^93^ The efficacy of IP-G47Δ treatment could be enhanced when it is started early in the treatment process.^60^ Further studies are needed to determine whether G47Δ treatment stimulates innate immunity to enhance antitumor effects.

Adenoviruses have been attractive candidates for PM treatment because of a high genetic stability and low degree of integration of their DNA with the host genome. A review by Sintya et al. showed that tropism of adenoviruses depends on the host cell receptors to which they can attach, and it can be further enhanced with different techniques.^94^ Because adenoviruses can be easily produced in high concentrations, they can be used in gene and cancer therapies, as well as in vaccine development.^95^ Recently, more attention has been paid to combination therapies using adenoviruses and other oncolytic viruses with a chemotherapeutic agent (e.g., paclitaxel). The Phase III PHOENIX-GC clinical trial suggested that IP paclitaxel could benefit patients with gastric PM.^49^ These findings encouraged further studies to determine whether the addition of IP oncolytic viral therapy to paclitaxel could be effective in gastric PM. OBP-301 is an oncolytic adenovirus, which drives the *E1A* and *E1B* genes for viral replication under control of the human telomerase reverse transcriptase (hTERT). By enhancing E1A-mediated cell-cycle mobilization and activating quiescent gastric cancer cells from the G0-G1 phase to the S phase, OBP-301 efficiently kills gastric cancer cells with elevated telomerase activity.^61^ The oncolytic adenovirus OBP-401 was generated by modifying OBP-301 with a GFP gene. An evaluation of a combination of OBP-401 and paclitaxel was conducted on selected human gastric cancer cell lines and a xenograft PM model (IP administration).^62^ OBP-401 combined with paclitaxel synergistically reduced the viability of human gastric cancer cells and increased the proliferative ability of the virus in cancer cells more than either OBP-401 or paclitaxel alone, significantly reducing the volume of malignant ascites and inhibiting the growth of the metastatic peritoneal tumor. The combination therapy also increased induction of the mitotic catastrophe, resulting in accelerated autophagy and apoptosis.^62^ Another variant of OBP-301, OBP-702, was used in a gene therapy trial against cancer-associated fibroblasts (CAFs) and PM of gastric cancer. A higher level of CAFs in any cancer tumors is associated with a poor prognosis.^63^ Through the modulation of CAFs and exogenous tumor-suppressor p53 overexpression, OBP-702 demonstrated significant antitumor effects against PM. In an MKN45-Luc xenograft mouse model, combined therapy of IP-administered OBP-702 and paclitaxel significantly suppressed IP tumor growth compared to control or monotherapy with either OBP-702 or paclitaxel.^96^ Combination therapy with oncolytic virus and paclitaxel may offer a novel and more effective treatment option for gastric PM. Under the control of a tumor-specific hTERT-cytomegalovirus promoter, another oncolytic adenovirus, Ad/TRAIL-E1, showed promise for PM from gastric cancer.^64^ Ad/TRAIL-E1 expresses tumor necrosis factor-related apoptosis-inducing ligand (TRAIL) and *E1A* genes and, therefore, induces TRAIL-mediated apoptosis in gastric cancer cell lines, but not in normal cells. IP-administered Ad/TRAIL-E1 significantly inhibited PM and increased the survival of mice without causing long-term toxicity.

Gene therapy seems a promising avenue to extending the survival time of patients with advanced stages of other GI cancers. Given that ∼30% of sporadic CRCs are caused by the inactivation of the tumor-suppressor gene p14ARF,^97^ the adenoviral construct of the human p14ARF gene (Ad-hARF) was tested for treating PM from CRC. p14ARF activates p53 and targets oncogenic C-terminal-binding proteins for degradation as part of its tumor-suppressor function.^65^^,^^66^ A single IP dose of Ad-hARF suppressed peritoneal disease progression in certain peritoneal xenograft models.^98^ Subsequently and importantly for adenoviruses, treatment with 6 lower doses of Ad-hARF had similar outcomes as with the single-dose regimen. Thus, for tumors in which the *ARF* gene has been inactivated, IP administration of Ad-hARF may be an effective method of treating PM.^98^

Rhabdoviruses show unique efficacy as boosting agents. The specific tropism of rhabdoviruses for PM is an area of ongoing research. They can break local immunosuppression, and their infection also leads to expanding secondary T cells by infecting splenic B cells, which present antigens to dendritic cells.^99^ Several viruses from the Rhabdovirus family have been studied for peritoneal delivery, including the vesicular stomatitis viruses (VSV) M51R and Maraba MG1, carrying mutations that can increase the selectivity of each virus toward tumor cells and reduce replication in healthy tissue. Through mutations in the matrix protein, which plays a major role in viral assembly and inhibits antiviral protein production in infected cells, VSV can become more selective for tumor cells.^67^^,^^68^ For example, IP delivery of M51R VSV in the mouse model inhibited the growth of CRC PM, and the survival was improved compared to controls.^69^^,^^70^ It was also observed that M51R VSV altered peritoneal cytokine profiles, with significant decreases in monocyte chemoattractant protein-1 and interleukin-6 (IL-6) levels.^69^ Another treatment of PM from CRC focused on the IP administration of autologous tumor cells in a murine model.^100^ MG1-IL-12-infected cell vaccine (ICV) stimulated recruitment of natural killer cells to the peritoneal cavity, and survival was longer for mice treated with MG1-IL12 ICV compared to MG1 ICV. Moreover, a long-lasting immunity developed in cured mice.^100^

Orthopoxviruses, belonging to the *Poxviridae* family, include vaccine viruses and the chimeric orthopoxvirus CF33, which has been recombined among 9 different orthopoxvirus species and strains. To enhance the effects of an oncolytic orthopoxvirus, CF33, the virus was armed with human sodium iodide symporter and anti-programmed death ligand 1 (PD-L1) antibody (CF33-hNIS anti-PD-L1).^101^ Infected cells with CF33-hNIS (human sodium iodide symporter) anti-PD-L1 produced anti-PD-L1 antibodies, which blocked PD-1–PD-L1 interaction and enhanced local antitumor immunity. Results showed that in the mouse model, IP delivery of CF33-hNIS anti-PD-L1 effectively reduced peritoneal tumor burden and improved survival and efficacy compared to intravenous (IV) administration.^101^

The vaccinia virus (vv) brings several additional unique advantages: it directly lyses the cell; it interferes with tumor angiogenesis; it decreases blood flow to tumor cells; and, ultimately, it causes hypoxia in tumor cells.^102^^,^^103^ These advantages suggest that vv can effectively modify the tumor microenvironment. It also activates the immune system to recognize and destroy cancer cells via various tropism mechanisms. Researchers examined the therapeutic efficacy of the genetically modified vv GLV-1h153 for the treatment of tumor cells, which efficiently regressed gastric cancer and permitted deep-tissue imaging.^104^ Orthotopic CRC-PM xenografts treated with IP-delivered GLV-1h153 significantly reduced tumor burden and could be efficiently monitored with computed tomography. The findings suggested that GLV-1h153 may benefit the treatment of CRC PM, thus offering another means for detecting viral distribution and tumor location noninvasively.^105^ Furthermore, in animals bearing peritoneal MC38 CRC, a novel oncolytic vv expressing the superagonist IL-15, a fusion protein of IL-15 and IL-15R-α(vvDD-IL15-Ra, IV administration) significantly reduced tumor growth and extended survival.^71^ The effect was mediated by the adaptive antitumor immunity provided by CD8^+^ T cells. A single treatment of vvDD-IL-15-Ra resulted in mice resistant to rechallenge with tumor cells.^71^ Furthermore, a superagonist of IL-15 also enhanced the antitumor activity of PD-1 blockade.^72^ The combination of an anti-PD-1 antibody and oncolytic immunotherapy led to CRC tumor regression and prolonged survival of mice more than either anti-PD-1 or vvDD-IL-15-Ra alone.^71^ Another vv, mJX-594, armed with granulocyte-macrophage-colony-stimulating factor (GM-CSF) also had higher anticancer immunity when combined with the immune checkpoint inhibitors anti-PD-1, anti-PD-L1, and anti-LAG3 when used to treat CRC (MC38 cells, mouse model, IP delivery), PM, and malignant ascites. The combination of mJX-594 and anti-VEGFR2 or immune checkpoint inhibitors resulted in the complete remission of peritoneal malignant ascites in some mice.^73^

Patients with PM of various origins, including gastric cancer, were studied in the Phase I clinical trial for the IP administration of oncolytic vv GL-ONC1.^106^ Nearly 90% of patients demonstrated efficient IP infection and replication of GL-ONC1, as well as subsequent oncolysis. The treatment was well tolerated with no dose-limiting toxicities observed, and no maximum tolerated dose was reached after IP administration.^106^ According to the study conducted with the JX-594 vv Pexa-Vec, oncolytic therapy can also be beneficial as a neoadjuvant for PM.^107^ Patients with CRC liver metastases received a single IV infusion of Pexa-Vec before surgical resection that led to tumor necrosis. Moreover, the plasma compartment within peripheral blood enabled the delivery of Pexa-Vec to the tumor, which promoted innate immunity against the tumor and resulted in long-term anticancer T cell responses. The tumor specificity of Pexa-Vec is enhanced by the deletion of thymidine kinase, an enzyme of the DNA precursor pathway expressed in high levels during malignant growth.^108^ In addition, Pexa-Vec expresses GM-CSF, which stimulates the proliferation and differentiation of myeloid precursor cells, as well as recruitment, maturation, and stimulation of dendritic cells, which enhance antitumor immunity.^109^ These results support the development of preoperative treatments with Pexa-Vec. The combination of Pexa-Vec and sorafenib, however, failed to successfully treat advanced hepatocellular carcinoma in a randomized Phase III clinical trial (this study was registered at Clinicaltrials.gov: NCT02562755).

In summary (Table 1), the most recent findings on oncolytic viral therapies support the development of IP viral therapy for patients with PM. Increasing the effectiveness of cancer treatment (via stimulation of tumor cells’ apoptosis or immune response) can be achieved by combining these viruses with chemotherapeutic drugs or checkpoint inhibitors. Similarly, the introduction of genes with anticancer functions into the genome of oncolytic viruses can enhance the therapeutic effect. Further studies, especially of humans, are needed to determine the potential of oncolytic viruses in treating PM of GI origin.

#### Bacteria-based therapies

A promising alternative treatment option is bacterial therapy used as a delivery system for heterologous antitumor molecules. The advantages of bacterial use include low price, immunostimulatory activity (adjuvant), biosafe strains, low side effects, and the tendency to colonize the tumor microenvironment.^110^ Studies have demonstrated that some anaerobes such as *Salmonella typhimurium* have a natural preference for tumor tissues.^74^ *Salmonella* can accumulate more readily in tumor tissue than in normal tissue, which leads to increased tumor growth inhibition and lower toxicities. In addition, it can be used as live attenuated bacterial vectors to deliver therapeutic agents, such as cytokines, antiangiogenic agents, tumor antigens, apoptosis-inducing factors, small interfering RNAs (siRNAs), and so forth. Combined with other cancer treatments, its immunomodulatory ability can enhance therapeutic benefits by eliciting strong adjuvant activities.^111^ The major obstacle is the intrinsic pathogenicity of *Salmonella*, which may cause serious toxicities, especially after systemic infection. Therefore, it is necessary to attenuate such bacteria, for example, via auxotrophic mutations.

The *Salmonella typhimurium*-aroA-auxotroph mutant called S636 with endostatin-expressing plasmid (S636/pES) led to decreased tumor growth and prolonged survival compared to PBS or bacteria with empty plasmids in IP-treated mice bearing CRC cells.^112^ Within tumor tissues, *Salmonella* colonization and expression of antiangiogenic endostatin were associated with increased levels of apoptosis and suppression of tumor angiogenesis.

In a Phase I clinical trial, *Salmonella typhimurium* was modified to express IL-2 (*Salmonella*-IL-2) and was used to treat 22 patients with liver metastases from GI cancers.^75^ *Salmonella*-IL-2 showed no toxicity or side effects after oral administration. There was no evidence of a complete response or survival advantage, possibly because of underlying advanced metastatic disease.

Further research is needed to optimize the design of bacterial vectors to maximize their ability to colonize and accumulate in tumor tissues while reducing the adverse effects of *Salmonella* on healthy tissues and endotoxicity. In addition, research is needed to optimize plasmid delivery to enable the expression of target proteins under controlled conditions, so that the therapeutic functions of *Salmonella* can be maximized, and its toxic side effects minimized.

#### Cellular therapies

Cellular therapies focus on using living cells as a drug. Although examples include a variety of cell types, in PM the transfer of immune chimeric antigen receptor (CAR) T cells showed the greatest potential for improved survival. CAR T cells are an example of a cell-gene immunotherapy in which a triple-mutated, third-generation oncolytic HSV-1, G47Δ r (CAR), is genetically engineered into T cells to target specific surface antigens and destroy tumor cells. The chimeric character lies in having both an antigen-binding character and a T cell-activating character. In hematological malignancies, CAR T cell therapy has already shown promising results.^113^^,^^114^ However, in solid tumors, its efficacy is limited, primarily because of the barrier related to a cold tumor environment inhibiting delivery, penetration, and activity of CAR T cells. Here, we summarize the most recent efforts in CAR T cell therapies in PM with GI origin.

In gastric cancer, CLDN18.2 CAR T cells have been investigated in a Phase I clinical trial that showed an overall response rate of 49% and a disease control rate of 73%, despite all of the patients developing an adverse event (predominately leukopenia).^115^ A wide variety of cancers, including PM from GI (particularly CRC) cancers, overexpress the epithelial-cell adhesion molecule (EpCAM) CD326.^116^ The EpCAM receptor has shown to be a promising therapeutic target in PM treatment in the trials that used catumaxomab immunotherapy.^117^^,^^118^ A new, third generation of anti-EpCAM-CAR T cells constructed to target EpCAM^+^ tumors exploited the potent antitumor activity of CAR T cells.^76^ The anti-EpCAM-CAR T cells showed controlled cytolytic activity as well as temporary expression of CAR T cells, which is relevant from a safety standpoint since EpCAM is also expressed on normal epithelium. Their repeated IP injections delayed disease progression in mice bearing peritoneal CRC xenografts, and lentiviral-transduced CAR T cells killed EpCAM^+^ human CRC cells *in vitro*.^76^ This therapy is being investigated in a clinical trial (this study was registered at Clinicaltrials.gov: NCT03563326).

The carcinoembryonic antigen (CEA) represents another adhesion molecule that can promote the aggregation and invasion of tumor cells. CEA issued as a marker of GI tumors, which can be useful for indicating metastases.^119^ CEA is highly expressed in CRC tissues and serum, whereas in normal adult tissues, except for the GI tract, it is not detected.^119^ It is interesting that the antigens are expressed on the luminal side of normal cells, making it invisible to immune cells. This feature suggested that CEA could be a suitable target for CAR T cell therapy. A Phase I clinical trial evaluated CAR T cell therapy for patients with metastatic CRC with overexpressed CEAs via IV infusion in escalating doses.^77^ CEA-CAR T cell therapy stabilized the disease in 70% of patients who developed progressive disease after previous treatments.^77^ Ongoing Phase I clinical trials include NCT05240950 and NCT04513431 (these trials are registered at Clinicaltrials.gov).

Another Phase I clinical trial used CAR T cells targeting CEA to treat patients with CEA^+^ liver metastasis from gastric, CRC, or ampullary origin.^78^ CEA-CAR T cells with or without the IL-2 cytokine were administered via hepatic artery infusions. At 23 months following infusions, 1 patient remained stable. In 5 other patients, the disease progressed. In combination with systemic IL-2, anti-CEA-CAR T cell therapy led to the enhancement of responses, with CEA levels reduced by 37% from the baseline. Moreover, elevated serum of interferon-γ levels (IFN-γ; IFN-γ regulates the immune response through cell signaling) was associated with IL-2 administration and decrease in CEA. Additional research efforts are needed to prove its efficiency. Recent studies have shown that IL-10 blockade also enhanced the antitumor efficacy of anti-CEA-CAR T cells in CRC liver metastases.^79^ Two Phase I clinical trials with CEA-expressing liver metastases have been completed: NCT02416466 and NCT02850536 (these trials are registered at Clinicaltrials.gov).

In the mouse model discussed by Katz et al.,^80^ an IP infusion of CAR T cells targeting CEA was found to be superior to the systemic administration of CAR T cells as a treatment for PM. IP injection of CAR T cells resulted in a greater reduction of tumors compared to IV infusion. Furthermore, it prevented recurrence and extraperitoneal tumor growth in the host. The myeloid-derived suppressor cells (MDSCs) and regulatory T cells (Tregs) are 2 immunosuppressive cell types in the metastatic solid tumor microenvironment that inhibit CAR T cells.^120^^,^^121^ In the next step, the combined IP-CAR T cell treatment with anti-MDSC and anti-Treg antibodies showed further improvement in efficacy against PM.^80^ The Phase I clinical trials using targeting the PD-L1 are currently recruiting (NCT05477927 and NCT04684459; these trials are registered at Clinicaltrials.gov).

In treating solid tumors, one of the main challenges is the presence of components of the tumor microenvironment that inhibit CAR T cell function and act as a physical and biochemical barrier to the tumor.^121^ Therefore, to improve outcomes, it is important not only to increase the efficacy, penetration, and migration of CAR T cell therapy but also to combine it with other strategies aimed at breaking or remodeling the tumor microenvironment barrier and preventing immunosuppression.

#### Nanotherapeutics and hydrogels

Nanoparticles are particles with diameters of 1–100 nm, with some formulations used in PM reaching 1,000 nm. Nanoparticles are excellent candidates for PM treatment because of their size, versatility, and ability to modify their surfaces.^122^ Nanoparticles can be used in a wide range of applications, including drug delivery, imaging, cancer detection, and energy production. Nanocarriers, nanoparticles acting as the carries of therapeutic agents, have been reviewed by Dakwar et al.^122^ Here, we provide a summary of nanoparticles that have recently been used in the treatment of PM. The use of nanoparticles as a delivery system for RNAi, which is a promising therapeutic agent for incurable cancers, has received considerable attention because of the ability of RNAi for posttranscriptional gene silencing.^123^ Therefore, a novel RNAi-based therapeutic nanoconstruct, DFP-10825, has been investigated.^124^ DFP-10825 consists of cationic liposomes and chemically synthesized short hairpin RNA (shRNA) against thymidylate synthase, a key enzyme for DNA synthesis and repair in cancer cells. In mice with gastric PM xenografts, IP administration of DFP-10825 showed promising results.^88^ Without causing severe side effects (because of very limited distribution to blood circulation), the drug inhibited tumor growth and prolonged survival time. Notably, DFP-10825 showed greater growth-inhibitory effects than conventional S1 treatment (5-FU prodrug tegafur, gimeracil, and oteracil) on tumors disseminated in peritoneum. The drug also remained in the peritoneal cavity over 72 h and selectively accumulated in disseminated tumors. With linTT1 tumor-penetrating peptide targeting p32, IP-administered nanoparticles demonstrate better tumor selectivity, leading to improved anticancer effects.

Another approach stimulating an immune response associated with the release of a large amount of tumor antigen includes thermal ablation. Although tumor ablation alone is not sufficient to induce a potent antitumor-immune response in patients,^125^ its combination with other strategies, such as nanoparticles, showed promising results in clinical trials.^126^ Superparamagnetic iron-oxide nanoparticles (SPIONs) have been used in cancer treatment and diagnosis because of their magnetic properties, chemical stability, and biocompatibility.^127^ Carboxydextran-coated SPIONs, administered IP, were used as magnetic nanoparticles in combination with an alternating magnetic field.^89^ Combined, they led to magnetic hyperthermia, and, as a result, PM were inhibited in the xenograft mice model.^89^ After magnetic field application, the magnetic nanoparticles accumulated in tumor tissues and generated heat, enabling thermal ablation and the destruction of cancer cells. In the same study, cell damage caused by magnetic hyperthermia using 7 nm of SPIONs was also effective on CRC and pancreatic cancer cell lines.^89^ However, magnetic hyperthermia did not completely eradicate the disseminated cancer cells, and malignant ascites developed. Furthermore, other studies have shown that *in situ* tumor ablation combined with locally injected nano-adjuvants (Toll-like receptor agonists PLGA-R837 and poly (lactic-co-glycolic acid)-monophosphoryl lipid A [PLGA-MPLA]) and anti-cytotoxic T lymphocyte-associated protein 4 (CTLA-4) blockade could inhibit metastasis and tumor recurrence in the CRC-PM mice model.^90^ PLGA-R837 and PLGA-MPLA nanoparticles activated dendritic cells, which are necessary for T cells to reject distant tumors, whereas anti-CTLA-4 antibody inhibited immunosuppressive Treg cells.

Treatments based on nanoparticles for PM with GI origin have shown promising results in clinical trials. Further research is needed to optimize nanoparticle design, determine the most effective drug combinations, and assess the long-term safety and effectiveness of these treatments.

#### Biologics and other approaches

In the quest for better treatments for PM, in addition to improvements in drug penetration into PM tumors with CAR T cells or viruses, scientists are investigating the molecular mechanisms that underlie the peritoneal dissemination of GI cancers and ways to address them.

Synaptotagmins (SYTs) are membrane-trafficking proteins that have 17 different isoforms. Three of them in particular, isoforms VII, VIII, and XIII, have been found to play an important role in the onset and progression of gastric, colon, and other cancers.^128^ This makes them a promising therapeutic target for treating PM. Studies showed that synaptotagmin XIII (*SYT13*) plays a role in the spread of gastric cancer and among patients with recurrent or PM expressing significantly higher levels of *SYT13* than those in stage I.^81^ IP administration of SYT13-specific siRNA, which inhibits *SYT13* expression in gastric cancer cells, inhibited peritoneal nodule growth and prolonged survival in a mouse model.^81^ SYT13-targeting antisense oligonucleotides (ASOs) modified with amido-bridged nucleic acids (AmNAs) were further investigated.^82^ IP administration of AmNA-modified anti-SYT13 ASOs inhibited the formation of peritoneal metastatic nodules and significantly prolonged survival in mice xenografted with gastric PM. Also, the expression levels of synaptotagmin VIII (SYT8) were higher in gastric cancer tissues of patients with peritoneal recurrence or metastasis.^83^ Synaptotagmin VII (SYT7) may also become a therapeutic target since it was found to be significantly associated with hepatic metastasis, recurrence, and poor prognosis in primary gastric cancer tissues.^129^ Furthermore, increased expression of SYT7 is associated with a greater pathological stage of CRC.^130^

The phosphoglycerate kinase 1 (PGK1) gene expression was shown to be significantly increased among patients with peritoneal dissemination of gastric tumors.^131^^,^^132^ In addition, PGK1 promotes chemotherapy resistance. Silencing PGK1 by IP delivery of shRNA with an adenoviral vector combined with 5-FU decreased the gastric PM number and weight in the PM mouse model more effectively than 5-FU alone.^84^ However, systemic toxicity remained present.

Heme oxygenase-1 (HO-1) is also thought to play a role in gastric cancer metastasis,^133^ and zinc protoporphyrin IX (ZnPPIX) has been identified as an inhibitor of HO-1.^134^ In a murine gastric PM xenograft model, IP ZnPPIX administration reduced the number, size, and weight of peritoneal metastatic nodules,^85^ suggesting that ZnPPIX may be useful as an antiangiogenic and anticancer agent with no apparent side effects.

An alternative approach involves altering the tumor microenvironment. Specifically, by the reduction of collagen fibers (a key component of the extracellular matrix), drug penetration and delivery to tumors can be improved.^135^ The main concern is the potential toxicity caused by the breakdown of collagen in healthy tissues. Nonetheless, the IP administration of collagenase to the CRC-PM murine model reduced tumor volume macroscopically and, when combined with IP mitomycin, resulted in an even greater volume decrease.^86^ In terms of safety, the animals did not show any abnormalities during the 8-week follow-up period. However, it was observed that high concentrations of collagenase caused local toxicity (thinning of bowel muscles).^86^ Moreover, tumor-penetrating peptides, such as iRGD (a cyclic 9-amino acid peptide CRGD[K/R]GP[D/E]C including RGD motif) or linTT1 (AKRGARSTA), also enhance the delivery of IP drugs, improving their effectiveness.^87^^,^^136^ The combination of iRGD and doxorubicin inhibited massive peritoneal tumor growth and reduced systemic toxicity in a PM-murine model (with IP administration).^87^ With linTT1, a peptide whose homing receptor is p32, iron-oxide nanoworms and the proapoptotic peptide D(KLAKLAK)2 were combined into linTT1-D(KLAKLAK)2-nanoworms. This combination showed p32-dependent cytotoxicity *in vitro* on gastric, CRC, and ovarian cancer cell lines.^136^

Overall, different agents show potential to support chemotherapy outcomes by improving drug delivery and inhibiting factors that contribute to metastasis. This brings hope that they can improve the results of treating PM with GI origin in the clinic in the very near future.

## Conclusions and future directions

PM from GI cancers continue to challenge clinical oncologists. Multimodal therapies, including CRS with HIPEC and systemic chemotherapy, still lead to morbidity and increase mortality rates. With novel practices developing (e.g., PIPAC), these treatments are limited to a small number of oncology centers nationwide and worldwide. More clinical trials are needed to fill the gaps between current approaches: systemic therapy and IP treatments.

We focused on the emerging novel therapies identified to support treatment and outcomes of PM. Most of the results, although preliminary and preclinical in murine models, are promising. Their continued development brings hope to further affect the outcomes of the PM patients and their caregivers in the near future. Continued integrated and interdisciplinary initiatives are required to analyze and merge several types of data (e.g., clinical, molecular, imaging) to better select patients and identify treatments. We foresee that developing computational tools, especially artificial intelligence-enabled models, will provide additional insights and identify patterns in the data, detect mutations with greater sensitivity, and help transfer information between multiple studies. Moreover, computational tools will augment the identification of novel targets for drug development and treatment optimization.
